# Prognostic and predictive value of tumor-infiltrating lymphocytes for clinical therapeutic research in patients with non-small cell lung cancer

**DOI:** 10.18632/oncotarget.7282

**Published:** 2016-02-09

**Authors:** Dong-Qiang Zeng, Yun-Fang Yu, Qi-Yun Ou, Xiao-Yin Li, Ru-Zhi Zhong, Chuan-Miao Xie, Qiu-Gen Hu

**Affiliations:** ^1^ Department of Radiology, Affiliated Shunde First People's Hospital of Southern Medical University, Foshan, Guangdong, P. R. China; ^2^ School of Basic Medical Sciences, Southern Medical University, Guangzhou, Guangdong, P. R. China; ^3^ Department of Ultrasound, Affiliated Shunde First People's Hospital of Southern Medical University, Foshan, Guangdong, P. R. China; ^4^ Imaging Diagnostic and Interventional Center, Sun Yat-sen University Cancer Center, State Key Laboratory of Oncology in South China, Collaborative Innovation Center for Cancer Medicine, Guangzhou, Guangdong, P. R. China; ^5^ Department of Medical Oncology, Nanfang Hospital, Southern Medical University, Guangzhou, Guangdong, P. R. China; ^6^ Guangdong Agricultural Reclamation Central Hospital, Affiliated Zhanjiang Cancer Hospital of Guangdong Medical University, Zhanjiang, Guangdong, P. R. China

**Keywords:** meta-analysis, molecular subtypes, non-small cell lung cancer, survival, tumor-infiltrating lymphocytes

## Abstract

**Background:**

Previous preclinical and clinical studies have shown that levels of tumor-infiltrating lymphocytes (TILs) significantly correlated with prognosis in non-small cell lung cancer (NSCLC), and survival after therapy; however, this finding remains controversial. We performed a meta-analysis, to evaluate, systematically, the clinical utilization of TIL subtypes in patients with NSCLC.

**Methods:**

The PubMed, ISI Web of Science, EMBASE, and Cochrane Library databases were searched to identify relevant studies. We pooled estimates of treatment effects, and hazards were summarized using random or fixed effects models to evaluate survival outcomes.

**Results:**

A total of 24 relevant studies involving 7,006 patients were eligible. The median percentage of lymph node positivity was 45.7% (95% confidence interval [CI], 37.1–56.4%). Pooled analysis shows that high levels of CD8^+^ TILs had a good prognostic effect on survival with a hazard ratio (HR) of 0.91 (*P* = 0.013) for death and 0.74 (*P* = 0.001) for recurrence, as did high levels of CD3^+^ and CD4^+^ TILs, with HRs of 0.77 (*P* = 0.009) and 0.78 (*P* = 0.005) for death, respectively. By contrast, high levels of FoxP3^+^ regulatory TILs had a worse prognostic effect for overall and recurrence-free survival, with HRs of 1.69 (*P* = 0.042) and 1.79 (*P* = 0.001), respectively. No individual study affected the results, and no publication bias was found.

**Conclusions:**

Our findings support the hypothesis that TILs could be a prognostic marker in NSCLC. High-quality randomized studies are needed to verify statistically the effect of TILs on prognosis in future research.

## INTRODUCTION

The host immune response has been demonstrated to be crucial for cancer invasion, progression, and subsequent metastasis [1, 2]. Furthermore, preclinical data suggest that a clinically relevant immunoadjuvant pathway can trigger an antitumor immune response, by causing an immunogenic cell death that allows antigen cross-presentation and activation of tumor-specific cytotoxic T cells [3, 4]. In particular, the intensity of the tumoral immune response influences the effectiveness of cancer therapy; levels of tumor-infiltrating lymphocytes (TILs), such as CD3^+^, CD4^+^, CD8^+^, and factor forkhead box P3 (FoxP3^+^) T cells were proved to be an expression of immune response that is associated with patient survival in a wide variety of tumor types [5–8]. Recently, new therapies that reactivate anticancer immune responses, for example, in breast cancer [9] and colorectal cancer [10], have entered clinical practice and have favorably improved outcomes. Similarly, TILs are a predictive biomarker of response to neoadjuvant chemotherapy in non-small cell lung cancer (NSCLC) [11].

In NSCLC, histological subtyping has been mostly relevant to clinicopathological variables for routine prognosis and treatment [12]. Tumor-infiltrating lymphocytes have been described to predominate in aggressive NSCLC diseases, such as adenocarcinoma [13, 14], squamous cell carcinoma [15], and large cell carcinoma [16]. Also reported is the high expression of TILs in the NSCLC, a subset with a known favorable prognosis [17, 18]. Nonetheless, the literature reveals that the characterization of TILs in the prognosis of patients with NSCLC is still debatable. The discordance in these results is mainly due to the substantial diversity in study design, assay methods, patient population, histological subtypes, immunological response involved, and methods and criteria used to qualify and quantify the immune response.

To date, a number of reports [13–16, 19–38] have informed that the subtype of TILs, and the density or location of the tumor are correlated with the survival of NSCLC. In an attempt to resolve such inconsistencies, as well as to uncover more accurate prognostic biomarkers, we conducted a meta-analysis of the existing data to evaluate, systematically, the clinical utility of TILs in NSCLC. Since many studies identified TILs by CD3^+^, CD4^+^, CD8^+^, and FoxP3^+^, we also analyzed the predictive value of these TILs subtypes, as well as of the ratios between these subsets in NSCLC.

## RESULTS

### Identification of eligible studies

**Figure 1:**
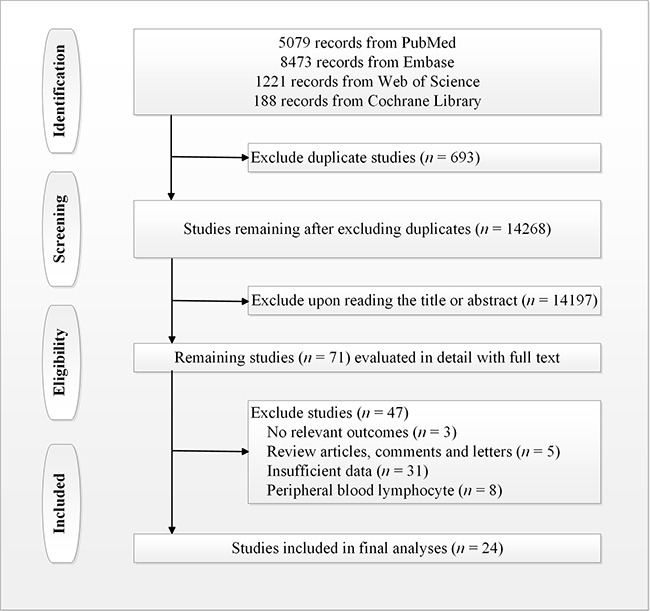
Study selection flow chart of eligible publications

A total of 14,961 potential articles were uploaded from PubMed, the ISI Web of Science, EMBASE, and the Cochrane Library. Of these, 14,133 articles were excluded because they did not satisfy all the inclusion criteria. The majority of these articles were excluded after reviewing the titles and abstracts because they were abstracts of non-outcome related studies, studies of other diseases, studies that were not related to TILs, studies of mouse models or cell lines, case or committee reports, review articles and meta-analyses, duplicate publications, or otherwise not related to studies evaluating the predictive roles of TILs in NSCLC. A total of 71 articles remained for full-text review. In the review, 47 articles were excluded for the following reasons: 5 articles were review articles, comments, or letters, 31 articles had insufficient data, 3 articles had no relevant outcomes, and 8 articles were studies of peripheral blood lymphocyte. Finally, 24 articles [13–16, 19–38] were included in the current meta-analysis. A summary of the article selection process is shown in Figure 1.

### Study characteristics

Characteristics of the 24 eligible studies are mainly summarized in Table 1. These studies were published between 2003 and 2015. Six studies [21, 25, 30, 35, 37, 38] concerned CD3^+^ T lymphocytes, six studies [14, 16, 19, 22, 28, 33] were of CD4^+^ T lymphocytes, and six studies reported on FoxP3^+^ T lymphocytes [13, 21, 27, 31, 33, 34]. The TILs ratio was reported in five studies, including both CD4^+^/CD8^+^ ratio [16, 22, 32] and FoxP3^+^/CD3^+^ ratio [13, 21], and 16 studies [14–16, 19, 20, 22–24, 26, 28, 29, 33, 35–38] considered CD8^+^ T lymphocytes, which was the most popular lymphocyte marker. The total sample size from all studies was 7,006, with a mean of 280 patients (95% confidence interval [CI], 170–412 patients) per study. Most of the studies were performed in Asia (*n* = 12). Others were conducted in Europe (*n* = 8), North America (*n* = 3), and South America (*n* = 1). Nine studies included fewer than 100 patients, while nine further studies enrolled >200 patients. The median percentage of positive lymph nodes, ranging from 26% to 100% in 18 reporting studies, was 45.7% (95% CI, 37.1–56.4%). The densities of TILs in intratumoral sites, stromal sites, and both sites combined were reported in 5 studies, 7 studies, and 14 studies, respectively. The most frequently used cut-off values for high versus low or positive verus negative density of TILs were the median (*n* = 8), and the staining score (*n* = 6).

**Table 1:** Characteristics of the included studies

No	Study/Year	Ethnicity	No. of Patient (male,%)	Tumor stage	Histologic subtype	%, Positive lymph nodes	TILs phenotype	Location	Definition of rich TILs	Outcomes
1	Wakabayashi 2003 [19]	Asia	178, (66%)	I:60%, II:13%, III:27%	ADC:53%, SCC:47%	36%	CD4^+^, CD8^+^	IS,SS	median	OS
2	Hiraoka 2006 [16]	Asia	109, (65%)	I:61%, II-III:39%	ADC:53%, SCC:36% LCC:7%, others:4%	29%	CD4^+^, CD8^+^, CD4^+^/CD8^+^ ratio	SS	≥5/HPF	OS
3	Ikeda 2006 [20]	Asia	83, (78%)	I:48%, II-III:52%	ADC:59%, SCC:28%, others:13%	45%	CD8^+^	IS+SS	TILs:≥50/HPF CD8:≥5/HPF	OS
4	Petersen 2006 [21]	North America	64, (53%)	I:100%	ADC:47%, SCC:34%, others:19%	NR	CD3^+^, FoxP3^+^, FoxP3^+^/CD3^+^ ratio	SS	median, Score ≥2	RFS
5	Al-Shibli 2008 [22]	Europe	335, (75%)	I:63%, II:27%, III:10%	ADC:28%, SCC:57%, LCC:9%	31%	CD4^+^, CD8^+^, CD4^+^/CD8^+^ ratio	IS+SS	CD4:IS:5%, SS:25% CD8:IS:5%, SS:50%	DSS
6	Kawai 2008 [23]	Asia	199, (70%)	IV:100%	ADC:67.3%, SCC:20.6%, others:12%	100%	CD8^+^	IS,SS	median	OS
7	Ruffini 2008 [24]	Europe	1290, (84%)	I:55%, II:21%, IIIa:16%	ADC:38%, SCC:43%, LCC:4%, others:15%	33%	CD8^+^	IS	1 ≥ positive staining in ≥20% of cells	OS
8	Al-Shibli 2010 [25]	Europe	335, (75%)	I:63%, II:27%, III:10%	ADC:28%, SCC:57%, LCC:9%	31%	CD3^+^	IS+SS	IS: ≥1%, SS: ≥50%	DSS
9	Dai 2010 [26]	Asia	99, (80%)	I:35%, II:20%, III:34%, IV:10%	ADC:45%, SCC:51%, LCC:4%	40%	CD8^+^	IS+SS	median	OS
10	Shimizu 2010 [27]	Asia	100, (60%)	I:68%, II:14%, III:18%	ADC:69%, SCC:31%	26%	FoxP3^+^	IS+SS	HPF ≥3	RFS
11	da Costa Souza 2012 [28]	South America	65, (60%)	I:31%, II:51% III:19%	ADC:58%, SCC:31%, LCC:11%	40%	CD4^+^, CD8^+^	IS+SS	CD8 >1.8%, CD4 >16.1%	OS
12	Ilie 2012 [29]	Europe	632, (74%)	I:39%, II:29%, III:27%	ADC: 55%, SCC: 33%, LCC:4%	NR	CD8^+^	IS	median	OS
13	Kayser 2012 [30]	Europe	232, (72%)	I:39%, II:26%, III:33%, IV:2%	ADC:32.3%, SCC:40.1%, LCC:27.6%	43%	CD3^+^	IS+SS	median	OS
14	Tao 2012 [31]	Asia	87, (64%)	NR	ADC:70%, SCC:23%, LCC:4%, others:3%	29%	FoxP3^+^	SS	HPF ≥25	OS
15	Hald 2012 [32]	Europe	55, (69%)	I:13%, II:36%, III:51%	ADC:29%, SCC:60%, LCC:11%	75%	CD4^+^/CD8^+^ ratio	SS	CD4:IS:5%, SS:25% CD8:IS:5%, SS:50%	OS
16	Suziki 2013 [13]	North America	956, (38%)	I:100%	ADC:100%	70%	FoxP3^+^, FoxP3^+^/CD3^+^ ratio	SS	Score ≥2	RFS
17	Hasegawa 2014 [33]	Asia	67, (66%)	I:70.2%, II:10.5%, III:19.3%	ADC:67.2%, SCC:23.8%, others:9%	75%	CD4^+^, CD8^+^, FoxP3^+^	IS+SS	≥3/HPF	RFS
18	Tao 2014 [34]	Asia	64, (84%)	II:17%, III:83%	ADC:48%, SCC: 45%, others:7%	30%	FoxP3^+^	IS+SS	median	OS, RFS
19	Djenidi 2015 [35]	Europe	191, (51%)	I:100%	ADC:45.5%, SCC:41.9%, others:12.6%	NR	CD3^+^, CD8^+^,	IS+SS	median	DFS
20	Donnem 2015 [36]	Europe	797	I:50%, II:34%, III:16%	ADC: 47%, SCC:43.8%, others:9.2%	NR	CD8^+^	SS	Low: ≤25%; high: >50%	OS,DSS, DFS
21	Schalper 2015 [37]	North America	a: 202, (51%)	I-II:68%, III-IV:32%	ADC:60%, SCC:17%, others:23%	NR	CD3^+^, CD8^+^	IS+SS	Score ≥3	OS
			b: 350, (88%)	I-II:60%, III-IV:40%	ADC:39%, SCC:48%, others:13%	NR	CD3^+^, CD8^+^	IS+SS	Score ≥3	OS
22	Kim 2015 [15]	Asia	331, (96%)	I:40%, II:36%, III:24%	SCC:100%	42%	CD8^+^	IS+SS	median	OS, DFS
23	Lin 2015 [14]	Asia	56, (37.5%)	NR	ADC:100%	NR	CD4^+^, CD8^+^	IS	Score ≥2	OS, RFS
24	Tian 2015 [38]	Asia	129, (30%)	I:37.2%, II:22.4% III:40.3%	ADC:37.21% SCC:47.29%, others:15.5%	48%	CD3^+^, CD8^+^	IS+SS	Score ≥3	OS

Abbreviations: ADC=adenocarcinoma; SCC=squamous cell carcinoma; LCC=large cell carcinoma; NSCLC=non-small cell lung cancer; HPF=high-power fields; IS=Intratumoral sites; SS=stromal sites; TILs=tumor-infiltrating lymphocytes; NR=no report; RFS=recurrence-free survival; DFS=disease free survival; OS=overall survival; DSS=disease specified survival; FoxP3+=regulatory T-lymphocytes expressing forehead box P3 protein; Schalper 2015a collection termed YTMA79, Schalper 2015b collection termed YTMA140.

### Overall meta-analysis

#### CD8^+^ T lymphocytes

**Figure 2:**
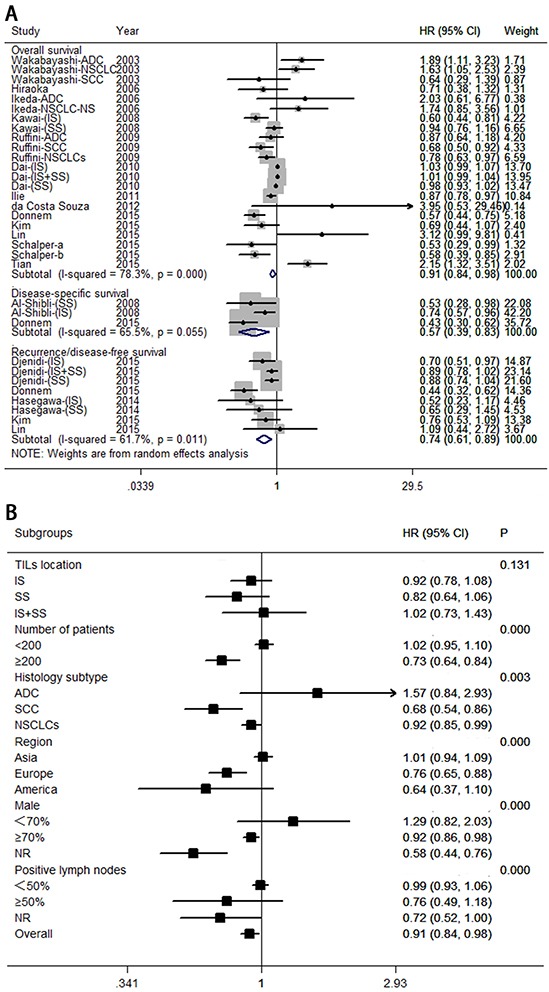
Forest plots of studies on CD8^+^ tumor-infiltrating lymphocytes **A.** CD8^+^ tumor-infiltrating lymphocytes and survival in NSCLC. **B.** CD8^+^ tumor-infiltrating lymphocytes are associated with overall survival among cancer patients, according to various characteristics. Hazard ratios and 95% confidence intervals for survival are associated with high versus low CD8^+^ counts; therefore a hazard ratio less than 1 represents a lower risk of death or progression associated with high CD8^+^ counts. ADC, adenocarcinoma; CI, confidence interval; HR, hazard ratio; IS, intratumoral sites; NR, not reported; NSCLC, non-small cell lung cancer; SCC, squamous cell carcinoma; SS, stromal sites; TIL, tumor-infiltrating lymphocyte.

**Figure 3:**
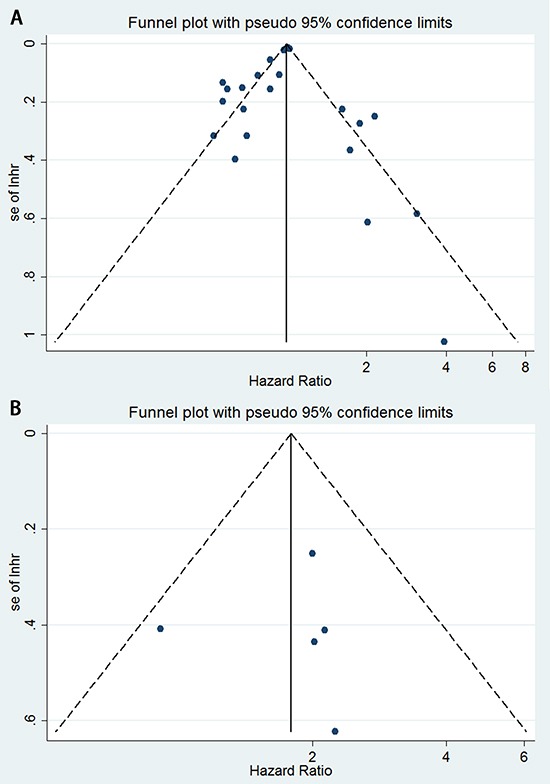
Funnel plots showing the associations between hazard ratios and standard error (se) for individual studies to assess publication bias **A.** CD8^+^ tumor-infiltrating lymphocytes for overall survival. **B.** FoxP3^+^ tumor-infiltrating lymphocytes for recurrence-free survival.

A total of 17 eligible studies were pooled for analysis of the density of CD8^+^ T lymphocytes as a prognostic and predictive marker in NSCLC; this analysis included 5,113 patients. Figure 2A indicates that high levels of CD8^+^ T lymphocytes indicate a good prognosis for overall survival (hazard ratio [HR] = 0.91; 95% CI, 0.84–0.98; *P*_HR_ = 0.013), disease-specific survival (HR = 0.57; 95% CI, 0.39–0.83; *P*_HR_ = 0.004), and recurrence- or disease-free survival (HR = 0.74; 95% CI, 0.61–0.89; *P*_HR_ = 0.001). Egger's test (*P* = 0.285) and Begg's test (*P* = 0.284), as well as a funnel plot (see Figure 3A), provided no evidence of publication bias for overall survival. However, significant heterogeneity was noted for overall survival (*I*^2^ = 78.3%, *P* = 0.000).

Meta-sensitivity analysis did not suggest undue influence of any single study. Therefore, we performed six predefined subgroup analyses to evaluate the effect of various clinical variables on pooled overall survival (see Figure 2B). These analyses revealed that high levels of CD8^+^ T lymphocytes were associated with improved overall survival in studies with large numbers of patients (≥200; HR = 0.73; 95% CI, 0.63–0.83), histology subtype (HR = 0.68; 95% CI, 0.54–0.86 for squamous cell carcinoma; and HR = 0.92; 95% CI, 0.85–0.99 for NSCLCs), European patients (HR = 0.76; 95% CI, 0.65–0.88). Any difference in these studies might be due to insufficient controls to confound for number of patients, histology subtype, region, percentage of men, or percentage of positive lymph nodes.

#### CD3^+^ T lymphocytes

**Figure 4:**
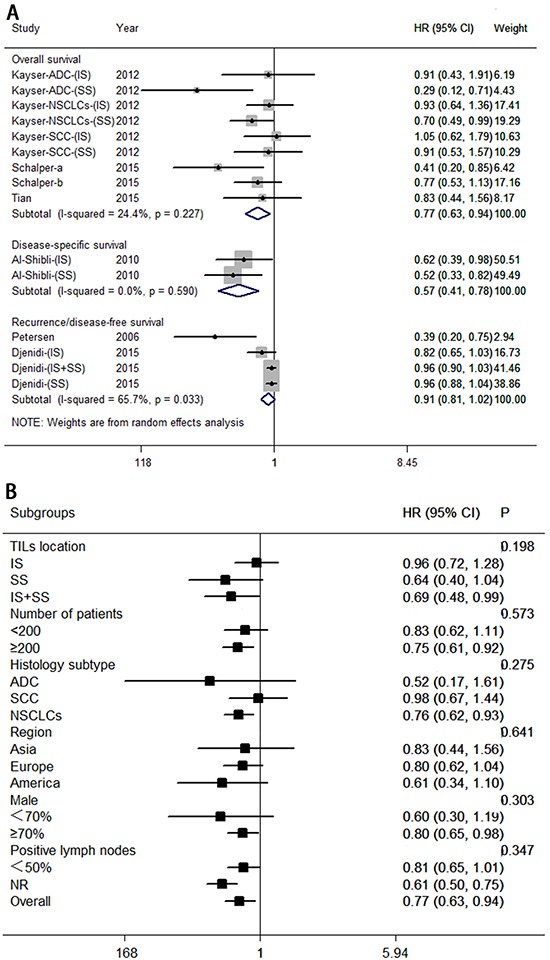
Forest plots of studies on CD3^+^ tumor-infiltrating lymphocytes **A.** CD3+ tumor-infiltrating lymphocytes and survival in NSCLC. **B.** CD3+ tumor-infiltrating lymphocytes are associated with overall survival among cancer patients according to various characteristics. Hazard ratios and 95% confidence intervals for survival are associated with high versus low CD3^+^ counts; therefore a hazard ratio less than 1 represents a lower risk of death or progression associated with high CD3^+^ counts. ADC, adenocarcinoma; CI, confidence interval; HR, hazard ratio; IS, intratumoral sites; NR, not reported; NSCLC, non-small cell lung cancer; SCC, squamous cell carcinoma; SS, stromal sites. TIL, tumor-infiltrating lymphocyte.

Six studies, involving 1,503 patients, investigated the association between the infiltration of CD3^+^ T lymphocytes and survival for patients with NSCLC. Pooled HRs and 95% CIs pointed to a survival advantage associated with high levels of CD3^+^ T lymphocytes, with improved overall survival (HR = 0.77; 95% CI, 0.63–0.94; *P*_HR_ = 0.009) and disease-specific survival (HR = 0.57; 95% CI, 0.41–0.78; *P*_HR_ = 0.001), but no significant correlation was found for recurrence- or disease-free survival (HR = 0.91; 95% CI, 0.81–1.02; *P*_HR_ = 0.115). These results are shown in Figure 4A.

We wondered whether a number of clinical variables would affect overall survival, and moderate heterogeneity was noted (*I*^2^ = 24.4%, *P* = 0.227). Therefore, subgroup analyses were carried out for overall survival (see Figure 4B). Exploratory subgroup analysis suggests that all patients benefit from high levels of CD3^+^ T lymphocytes with respect to location of both sites (intratumoral and stromal; HR = 0.69; 95% CI, 0.48–0.99); large number of patients (≥200; HR = 0.75; 95% CI, 0.61–0.92), histology subtype (NSCLCs; HR = 0.76; 95% CI, 0.62–0.92), percentage of men (≥70%; HR = 0.80; 95% CI, 0.65–0.98). There was no evidence of a difference in treatment effects between any of the subgroups.

#### CD4^+^ T lymphocytes

**Figure 5:**
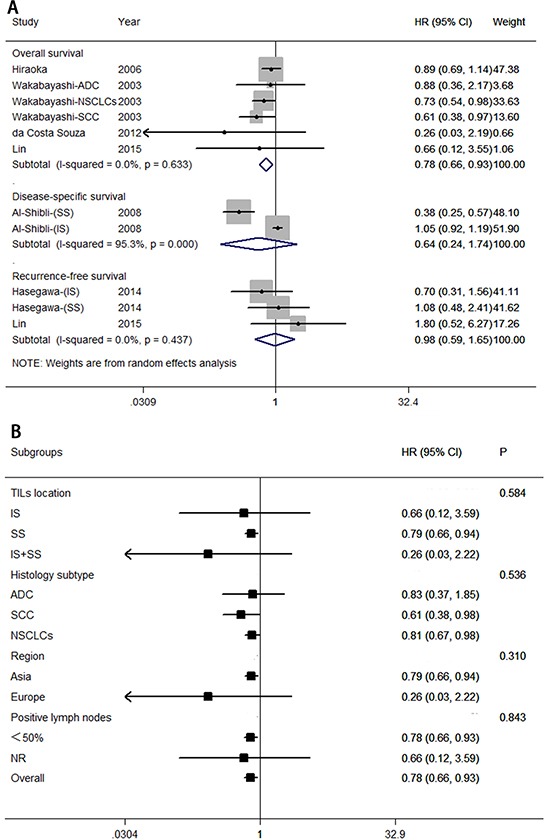
Forest plots of studies on CD4^+^ tumor-infiltrating lymphocytes **A.** CD4+ tumor-infiltrating lymphocytes and survival in NSCLC. **B.** CD4^+^ tumor-infiltrating lymphocytes are associated with overall survival among cancer patients according to various characteristics. Hazard ratios and 95% confidence intervals for survival are associated with high versus low CD4^+^ counts; therefore a hazard ratio less than 1 represents a lower risk of death or progression associated with high CD4^+^ counts. ADC, adenocarcinoma; CI, confidence interval; HR, hazard ratio; IS, intratumoral sites; NR, not reported; NSCLC, non-small cell lung cancer; SCC, squamous cell carcinoma; SS, stromal sites. TIL, tumor-infiltrating lymphocyte.

Six eligible studies provided estimates of HR and 95% CI for the association between density of CD4^+^ T lymphocytes and survival. Figure 5A indicated that a high level of CD4^+^ T lymphocytes was correlated with a good prognosis for overall survival (HR = 0.78; 95% CI, 0.66–0.93; *P*_HR_ = 0.005), but no significant difference was found for disease-specific survival (HR = 0.64; 95% CI, 0.24–1.74; *P*_HR_ = 0.386), and recurrence- or disease-free survival (HR = 0.98; 95% CI, 0.59–1.65, *P*_H*R*_ = 0.953).

We also carried out subgroup analyses to assess whether various clinical variables would affect overall survival (see Figure 5B). Exploratory subgroup analysis suggests that all patients benefit from high levels of CD4^+^ T lymphocytes with respect to TIL location (stromal sites; HR = 0.79; 95% CI, 0.66–0.94), histology subtype (HR = 0.61; 95% CI, 0.38–0.98 for squamous cell carcinoma; and HR = 0.81; 95% CI, 0.67–0.98 for NSCLCs), Asian patients (HR = 0.79; 95% CI, 0.66–0.94), or percentage of positive lymph nodes (<50%; HR = 0.78; 95% CI, 0.66–0.93). There was no evidence for a difference in treatment effect between any of the subgroups.

#### FoxP3^+^ Treg lymphocytes

**Figure 6:**
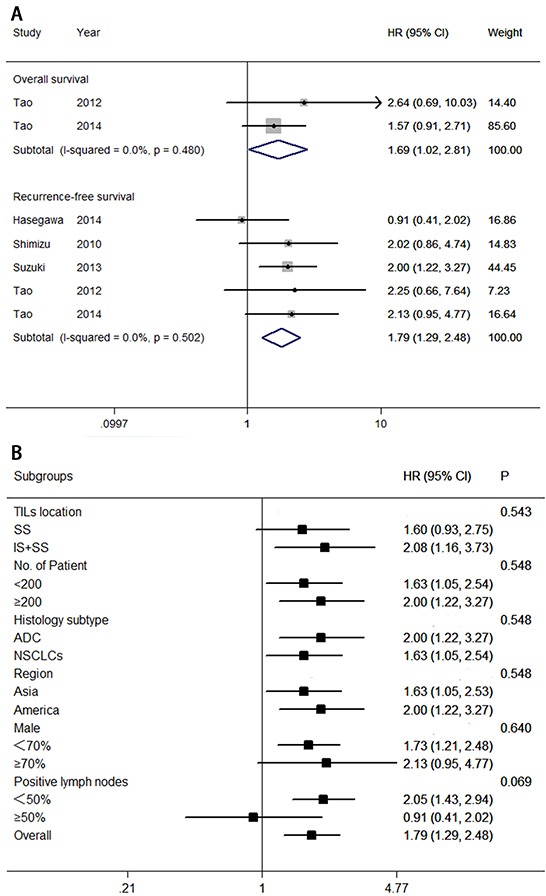
Forest plots of studies on FoxP3^+^ TILs **A.** FoxP3^+^ tumor-infiltrating lymphocytes and survival in NSCLC. **B.** FoxP3^+^ tumor-infiltrating lymphocytes are associated with recurrence-free survival among cancer patients according to various characteristics. Hazard ratios and 95% confidence intervals for survival are associated with high versus low FoxP3^+^ counts; therefore a hazard ratio less than 1 represents a lower risk of death or progression associated with high FoxP3^+^ counts. ADC, adenocarcinoma; CI, confidence interval; HR, hazard ratio; IS, intratumoral sites; NSCLC, non-small cell lung cancer; SS, stromal sites. TIL, tumor-infiltrating lymphocyte.

The value of FoxP3^+^ Treg lymphocytes for predictingsurvival was reported in six studies, involving 1,338 patients. Figure 6A indicated that low levels of FoxP3^+^ Treg lymphocytes correlated with a good prognosis for overall survival (HR = 1.69; 95% CI, 1.02–2.81; *P*_HR_ = 0.042), and recurrence-free survival (HR = 1.79; 95% CI, 1.29–2.48; *P*_HR_ = 0.001). Egger's test (*P* = 0.860) and Begg's test (*P* = 0.806), as well as a funnel plot (see Figure 3B), provided no evidence of publication bias for recurrence-free survival.

Subgroup analyses were also conducted to assess the potential correlation of various clinical variables with recurrence-free survival (see Figure 6B). Exploratory subgroup analysis suggested that all patients benefit from low levels of FoxP3^+^ Tre lymphocytes with respect to TIL location at both sites (intratumoral and stromal sites; HR = 2.08; 95% CI, 1.16–3.73), number of patients (HR = 2.00; 95% CI, 1.22–3.27 for ≥200; and HR = 1.63; 95% CI, 1.05–2.54 for <200), histology subtype (HR = 2.00; 95% CI, 1.22–3.27 for adenocarcinoma; and HR = 1.63; 95% CI, 1.05–2.54 for NSCLCs), Asian patients (HR = 1.63; 95% CI, 1.05–2.54), American patients (HR = 2.00; 95% CI, 1.22–3.27), percentage of men (<70%; HR = 1.73; 95% CI, 1.20–2.47), or percentage of positive lymph nodes (<50%; HR = 2.05; 95% CI, 1.43–2.94). There was no evidence of a difference in treatment effects between any of the subgroups.

#### Ratios between T lymphocyte subsets

**Figure 7:**
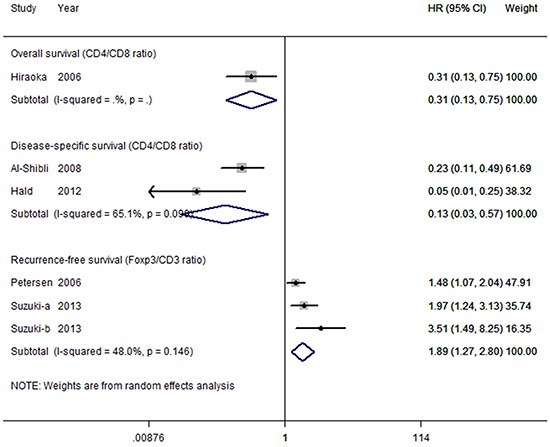
Forest plot of tumor-infiltrating lymphocyte ratios and survival in NSCLC Hazard ratios and 95% confidence intervals for survival are associated with high versus low tumor-infiltrating lymphocyte ratio counts; therefore a hazard ratio less than 1 represents a lower risk of death or progression associated with high tumor-infiltrating lymphocyte ratio counts. CI, confidence interval; HR, hazard ratio.

Relatively few studies evaluated the prognostic impact of TIL ratio subsets on survival. Thus, we only investigated the associations between survival and CD4^+^/CD8^+^ ratio, and between survival and FoxP3^+^/CD3^+^ ratio. All studies concerned with ratios were pooled in a forest plot (Figure 7). Surprisingly, pooled results for three studies reporting recurrence-free survival based on FoxP3^+^/CD3^+^ ratio were strongly significant (HR = 1.89; 95% CI, 1.27–2.80; *P*_HR_ = 0.002; *I^2^* = 48%), indicating that a high FoxP3^+^/CD3^+^ ratio is a risk factor for recurrence. Three studies observed that high CD4^+^/CD8^+^ ratio co-expression are favorable compared with low numbers of both cell types for overall survival (HR = 0.31; 95% CI, 0.13–0.75; *P*_HR_ = 0.009) and disease-specific survival (HR = 0.13; 95% CI, 0.03–0.57; *P*_HR_ = 0.007); however, a moderate to high heterogeneity was noted for disease-specific survival (*I^2^* = 65.1%, *P* = 0.146).

## DISCUSSION

In this meta-analysis, which included data of a cohort of 7,006 patients identified as having NSCLC from 24 studies, we provided quantitative estimates of the prognostic value of TILs in patients with NSCLC. We have demonstrated that comparing high and low densities of CD3^+^, CD4^+^, or CD8^+^ TILs alone in patients with NSCLC indicated that high densities of these subtypes of TILs alone could be a relatively pronounced predictive marker, with better associated outcomes than low infiltrate densities in terms of overall survival. Similarly high levels of CD3^+^ or CD8^+^ lymphocytes, or high CD4^+^/CD8^+^ ratios, were strongly independent prognostic biomarkers for disease-specific survival, but were used in relatively few studies. By contrast, low levels of FoxP3^+^ regulatory TILs or low FoxP3^+^/CD3^+^ ratio were found to correlate with a good prognosis for overall or recurrence-free survival. Data on NSCLCs were somewhat limited; thus, the analysis failed to demonstrate a disease-specific survival prognostic value for FoxP3^+^ regulatory TILs.

Since tumor-infiltrating immune cells have been shown to have prognostic value for several solid malignancies, immunotherapy has attracted much research interest. Historically, a number of researches have advocated, through their work in cancer, the use of three important parameters of TILs—subtype, density, and location—to predict clinical outcomes [7, 39, 40]. This is somewhat consistent with another study evaluating CD4^+^ and CD8^+^cells by chromogenic immunohistochemistry in patients with NSCLC. The authors of this study [16, 19] found an association between high levels of CD4^+^ T cells in cancer stroma and longer survival. However, these results were obtained from relatively small collections of samples from single institutions and without validation in an independent set. In our meta-analysis, data on patients with NSCLC were analyzed to assess the potential contributions of various clinical variables to survival outcomes. We investigated four markers of TILs and found that high levels of CD4^+^ lymphocytes in tumor-associated stroma to be significantly associated with survival. There is compelling evidence that this is due to the immunosuppressive effects of CD4^+^ T cells, which play a central role in orchestrating the immune response to lung cancer [41]. Interestingly, CD4^+^ lymphocytes in the stroma only, and not in intratumoral sites, were associated with death, emphasizing the importance of assessing the location of TILs within the tumor microenvironment. In fact, the significance of immune cells in the tumor stroma has been shown in NSCLC.

It is well known that patients with different subtypes of NSCLC have different responses, and we demonstrate that in addition to TILs, subtype, density, and location, a fourth characteristic of TILs—NSCLC subtype—is an important parameter. Stratified analysesrevealed that the survival of patients with squamous cell carcinoma has a positive association with high density of CD4^+^ or CD8^+^ TILs alone. Additionally, our analysis showed that high levels of FoxP3^+^ regulatory T cells are thought to play protumor roles, and their significant association with greater rates of recurrence has been shown for adenocarcinoma. FoxP3^+^ is a marker of regulatory T cells, a subset of TILs thought to play a major role in hampering antitumor immune response, and to represent a major cellular mechanism underlying immune evasion of lung cancer [41]. In patients with lung cancer, regulatory T cells are thought to play protumor roles and their association with worse prognosis has been demonstrated for all histologic types [42]. In addition to revealing prognostic value, this finding has significant implications for devising potential immunomodulatory therapy for patients with lung adenocarcinoma and squamous cell carcinoma; an intervention that decreases levels of FoxP3^+^ and increases levels of CD4^+^ or CD8^+^ TILs would likely to be beneficial. Furthermore, more well-designed clinical trials are needed to confirm the clinical prognostic utilization of TIL subtypes in patients with different subtypes of NSCLC.

In addition to the presence of different subsets of TILs, ratios some of these types of subset were also reported in previous clinical studies. We investigated the associations between NSCLC survival and CD4^+^/CD8^+^ ratio and between NSCLC survival FoxP3^+^/CD3^+^ ratio. Our analysis clearly demonstrated that a good response FoxP3^+^/CD3^+^ ratio was a risk factor for disease recurrence, while a good response CD4^+^/CD8^+^ ratio is favorable for survival, compared with low numbers of both cell types for overall and disease-specific survival. Additionally, a survival analysis by Kayser *et al.* [30] showed that high numbers of stromal CD4^+^/CD25^+^ T lymphocytes are of beneficial prognostic influence in patients with NSCLC, especially with adenocarcinomas. Previous experiments demonstrated that CD4^+^/CD25^+^ T lymphocytes have a regulatory function on tumor-reactive cytotoxic T lymphocytes and thereby successfully suppress lymphocytic tumor rejection [41, 43]. Ilie *et al.* [29] reported on the relationship between ratio of CD66b^+^/CD8^+^ TILs and survival. This study demonstrated that a high CD66b^+^/CD8^+^ ratio is an independent prognostic factor for a high rate of disease recurrence and death in patients with NSCLC. Considering the limited number of studies that reported the relationship between survival and ratios or changes in subtypes of TILs, more prospective studies are needed.

Beside the potential prognostication for NSCLC, we observed that TILs are a potential predictive biomarker for therapy. Liu *et al.* proved that the ratio of CD8^+^/FoxP3^+^ TILs independently predicted a good response to platinum-based chemotherapy for patients with advanced NSCLC [11]. Kawai *et al.* [23] reported that higher CD8^+^ infiltration within the tumor nest after platinum-based chemotherapy was strongly associated with better overall survival in patients with stage IV NSCLC. Tao *et al.* [34] demonstrated that a low density of FoxP3^+^ TILs indicated a better response to induction chemoradiation and better survival in locally advanced NSCLC, although the difference was not statistically significant, suggesting that FoxP3^+^ TILs might be a target for adjunct immunotherapy. Furthermore, cytotoxic CD8^+^ T cells are crucial in novel therapies targeting the immune system, e.g., by blocking CD8 T cell-related ligands (PD-L1 and PD-L2) and receptors (PD-1 and CTLA-4), antitumor immunity is enhanced in patients with various types of advanced solid tumors, including NSCLC [44]. These findings on TILs provided comprehensive information and a rationale for PD-1/PD-L1 pathway-targeted immunotherapy and other promising immunotherapy for patients with NSCLC. Based on these studies, the accumulation of CD8^+^ TILs and depletion of FoxP3^+^ TILs are thought to be favorable prognostic factors in patients with NSCLC, and these findings support the idea that augmentation of the local immune response might be a promising target for new immunotherapeutic approaches [45]. However, owing to insufficient data on the associations between levels of TILs and prognostic responses after systemic therapy, we did not find a statistically favorable survival outcome in pooled analyses. We believed that TILs could be monitored as a potential predictor for future therapies to treat NSCLC. High-quality randomized studies are needed to verify, statistically, the effect of TILs on prognosis in future NSCLC clinical therapeutic research.

The major strength of this study is that we have searched all published studies via electronic and hand searching that met the inclusion criteria and that no single study affected the results, and no publication bias was found in the survival panels, indicating that our main findings are robust. To the best of our knowledge, this meta-analysis is the first comprehensive assessment of the prognostic value of TILs for NSCLC, which may be useful for future research. Moreover, we conducted a stratified analysis for overall results based on location of TILs, number of participations, histology subtype, region, percentage of men, and percentage of positive lymph nodes, which could improve the reliability of the results and reduce the performance bias of the meta-analysis. Furthermore, the included studies, which were published between 2003 and 2015, provide accumulating evidence and a large sample size, which significantly increased the statistical power of the analysis to provide precise and reliable risk estimates. In addition, we dutifully preformed a broad search strategy for articles that considered the most frequently used T lymphocyte markers for NSCLC survival prognostication. Finally, the inclusion of studies from different countries suggests that the clinical utilization of TIL subtypes in patients with NSCLC is a global concern, with lung cancer accounting for more than one-quarter (27%) of all cancer deaths [46].

Despite these advantages, several limitations might be acknowledged in this meta-analysis. First, there is no international unification measurement to determine levels of TILs, and TIL location is also not assessed in a standardized manner; this increased the difficulty in performing the meta-analysis. Subsets of TILs from different locations, as well as subtypes of NSCLC should be investigated in future studies.

Second, all of the included studies were retrospective in design, with insufficient data, such as therapy details, tumor stage, cut-off point, smoking history, patient age, or molecular tumor alterations (e.g., epidermal growth factor receptor (EGFR), Kirsten rat sarcoma viral oncogene, or anaplastic lymphoma kinase), and thus the results could not be further stratified with other potential confounding factors that can affect the major outcomes. Moreover, further research on associations between TILs and clinicopathologic characteristics (e.g., PD-1 expression, PD-L1 expression), and more high-quality continuous treatment trials with TILs that might improve survival of NSCLC using immune-targeted chemotherapy or molecular targeted therapy, are needed to conform these results.

Third, in several studies, HRs for outcome measures were derived from Kaplan–Meier survival curves when not provided by the original studies directly; these would affect the level of evidence. Therefore, the facticity of the results might be influenced by this. Finally, further studies with better confounding factor adjustments are needed because data in the original publications could not be obtained, which might affect the risk estimates.

In conclusion, despite these limitations, we have demonstrated that TILs might serve as a robust marker for prognosticating the survival of patients with NSCLC, especially in TIL subtypes CD3^+^, CD4^+^, CD8^+^, and FoxP3^+^. Future well-designed clinical trials, especially randomized controlled trials, are required to confirm current findings and statistically verify the effect of TILs on prognosis in future clinical therapeutic research on NSCLC.

## MATERIALS AND METHODS

### Literature search

We followed the PRISMA guidelines (preferred reporting items for systematic reviews and meta-analyses statement) for this meta-analysis [47]. The PubMed, ISI Web of Science, EMBASE and Cochrane Library databases (updated to March 2015) were searched to identify relevant studies that investigated the predictive clinical outcome of TILs in NSCLC. The following terms were used: “Lymphocytes, Tumor-Infiltrating,” “T Lymphocytes,” “FoxP3-positive T lymphocytes,” “CD8-Positive T Lymphocytes,” “CD3-Positive T Lymphocytes,” “CD4-Positive T Lymphocytes,” “T lymphocytes,” “non-small cell lung cancer,” “Lung Adenocarcinoma,” and “Lung Squamous cell carcinoma.” No restriction was imposed on the search in terms of sample size, population, time period, language, or type of report. All eligible studies were retrieved, and the reference lists of the reviews or studies identified in the literature search were hand-searched for additional information when key information was missing.

### Inclusion criteria

Studies were included according to the following criteria: (1) the studies investigated the predictive clinical outcome of TILs (including CD3^+^, CD4^+^, CD8^+^, and FoxP3^+^ lymphocytes, and including ratios between these subsets) as a prognostic and predictive marker in patients with NSCLC, as identified by hematoxylin & eosin or immunohistochemistry staining, and which analyzed lymphocytes in intratumoral or stromal sites; (2) the studies were published as original full-text articles; (3) the studies reported prognostic information, including HRs for the relationship between TILs and tumor response outcome measures, including overall survival, recurrence- or disease-free survival, and disease-specific survival, or reported adequate data for the HRs to be computed. If duplicate data were presented in several studies, only the most recent and largest or most complete study was included.

### Data extraction and outcome measure

The information from the studies was independently extracted by two researchers (Dong-Qiang Zeng and Yun-Fang Yu) according to the inclusion criteria, and the data were checked by other investigators. Data were abstracted as follows: first author, year of publication, ethnicity, number of patients, percentage of men, tumor stage, histologic subtype, percentage of positive lymph nodes, TIL subsets, TIL locations, definition of high levels of TILs, and outcomes of univariate or multivariate analysis reported. However, as all of the included articles were retrospective, the quality of each study was not assessed.

The outcomes from univariate or multivariate Cox regression, that is, HRs and 95% CIs, were used for analysis. If both univariate and multivariate analysis for the same comparison were reported in studies, we only used the latter. When Kaplan–Meier curves were provided rather than the HRs, the HRs were calculated indirectly from the curves using the procedure proposed by Tierney *et al.* [48], which is based on the method reported by Parmar *et al.* [49]. The data which were collected were in accordance with the quality of meta-analyses statement. Since studies used different definitions for high and low levels of TILs, we considered the ratio of results between tumors with high levels of TIL expression versus those with low levels of or no TIL expression for each TIL subset. The reciprocals of HRs and CIs were taken to calculate the results the other way around, for studies that reported HRs for low versus high levels of TILs. To ensure the accuracy of the extracted information, other investigators who judged the inclusion and exclusion of the studies were blinded to the identity information of the studies. Disagreements on eligibility were resolved through discussion and consensus with other authors.

### Statistical analyses

For time-to-event data, we pooled estimated HRs, together with associated 95% CIs from the original articles. For overall results, *P* < 0.05 was considered statistically significant. *I*^2^ statistics [50] were applied to measure the heterogeneity of the studies and to provide a quantitative measure of inconsistency among studies. A random-effects model, the DerSimonian and Laird method [51], was utilized when *I*^2^ > 50% or *P* < 0.1; otherwise, a fixed-effect model, the Mantel–Haenszel method [52], was applied. When heterogeneity was observed, either subgroup or sensitivity analysis was performed, to assess the potential contributions of various clinical variables to the main outcome, while sensitivity analysis was performed by sequentially excluding studies in turn, to test the stability of the main results. Additionally, since we wondered whether study characteristics would affect study outcomes, subgroup analyses were carried out for the overall results. The potential publication bias was evaluated through visual inspection of a contour-enhanced funnel plot [53], Begg's test [54] and Egger's [55] unweighted regression tests. *P* < 0.05 indicates publication bias, and *P* > 0.05 indicates no bias. All tests were two-sided. Statistical analyses were calculated using STATA version 12. 1 (STATA Corporation, College Station, TX, USA). To ensure the reliability and accuracy of the results, two authors independently uploaded the data.
